# Concentration-dependent disturbances of digestive functions in house cricket (Insecta: Orthoptera) exposed to GO-AgNP composite

**DOI:** 10.1038/s41598-025-97589-w

**Published:** 2025-04-13

**Authors:** Reyhaneh Seyed Alian, Barbara Flasz, Andrzej Kędziorski, Magdalena Rost-Roszkowska, Katarzyna Rozpędek, Łukasz Majchrzycki, Maria Augustyniak

**Affiliations:** 1 Institute of Biology, Biotechnology and Environmental Protection, University of Silesia in Katowice, 40-007 Katowice, Poland; 2 Institute of Physics, Faculty of Materials Engineering and Technical Physics, Poznan University of Technology, Piotrowo 3, 60-965 Poznan, Poland

**Keywords:** *Acheta domesticus*, Food consumption and assimilation, Dead cells and ROS + cells, Gut histology, Digestive enzymes, Physiology, Biomarkers, Nanoscale materials

## Abstract

This study investigates the effects of graphene oxide (GO) and silver nanoparticle (AgNP) composite (GO-AgNP) on the digestive physiology and gut ultrastructure of *Acheta domesticus* (house cricket) during extended exposure. Various concentrations of GO-AgNP were tested to assess their impact on food consumption, assimilation, cell status (Dead Cells and ROS + cells), gut enzyme activity, and structural damage to gut cells. Concentration and exposure time had significant effects on oxidative stress, enzyme activity, and gut cell structure. The applied composite reduced cumulative food consumption and assimilation efficiency. Enzyme assays showed that lower concentrations enhanced carbohydrate-degrading enzyme activity, while higher concentrations inhibited protease activity. Histological analysis revealed structural damage to gut epithelial cells and signs of autophagy or necrosis at higher concentrations. These results suggest that GO and AgNPs contribute to oxidative stress, cell cycle disruption, and apoptosis, with AgNPs having a potentially stronger effect than GO. The disturbed enzyme activity may result from conformational changes caused by nanoparticle agglomeration. These findings underline potential risks associated with the environmental or agricultural use of GO-AgNP composites.

**Supplementary Information:**

The online version contains supplementary material available at 10.1038/s41598-025-97589-w.

## Introduction

The rapidly advancing field of nanotechnology continuously introduces new nanomaterials with structural and functional properties that render them desired in a wide array of applications from industry to everyday household life^1–5^. The widespread use of nanomaterials leads to increased environmental exposure to nanoparticles, affecting biota. Since they enter food webs and contaminate food products, there is an urgent need to identify their potentially deleterious effects, both in terms of acute and chronic exposure, encompassing a broad range of concentrations and durations^6,7^.

Graphene oxide (GO) is among the most attractive nanomaterials due to its unique properties, which make it valuable for applications in electronics, energy storage, materials engineering (synthesis of nanocomposites and coatings), optics, medicine, environmental technologies, and many other fields^8–11^. Similarly, silver nanoparticles (AgNPs) are among the most commonly used metallic nanoparticles, primarily because of their potent antimicrobial properties, optical characteristics, and excellent thermal and electrical conductivity^12–14^. While the harmful effects of GO and AgNPs have been relatively well-studied, their impact on the digestive functions of organisms remains an area with considerable room for further research^15–18^.

Recently, new composite nanomaterials have been synthesized based on GO and metal nanoparticles. They combine the properties of their constituents or exhibit novel features, further expanding their range of applications^19–22^. An example of such nanomaterial is the GO-AgNP composite, whose diverse applications have been clearly presented by de Medeiros et al.^23^. The application of GO-AgNP composites in medicine results from their unique antibacterial properties^24–27^. Additionally, their use has been proposed for various purposes, including drug delivery platforms, wound healing, bioimaging materials, and components in sensors for detecting various substances, such as glucose^28–30^. GO-AgNP composites are applied also in agriculture, plant protection, dye degradation, catalysis, electrochemical detection, and even environmental engineering^31–37^. However, using these GO-based nanomaterials raises the question of whether the GO and metal nanoparticles, as components of composite materials, interact within organisms in an additive, synergistic, or antagonistic manner.

Surprisingly, nanoparticles’ impact on organisms’ digestive functions has not been extensively studied. The available data on the activity of digestive enzymes are fragmentary, indicating the potential inhibition of certain digestive enzymes by some nanoparticles, but also, under certain conditions, their stimulation^38–45^. According to our previous studies, the effect of GO on digestive enzymes is moderate and depends on the concentration and type of enzyme studied. High concentrations of GO primarily stimulated the activity of amylase and lipase, while prolonged exposure inhibited protease activity. Notably, the effects described above were much more pronounced in the case of AgNPs^45^.

The impact of nanoparticles on digestive functions is multifaceted, potentially affecting both host enzymes, the gut microbiota and possibly nutrients absorption by the gut cells^17^. These impairments can result in nutrient and energy shortages and disturb proper development, growth, reproduction, and adequate stress responses. In one scenario these may lead to increased mortality of exposed organisms. However, there may be different scenario, particularly following lower exposure - an organism may have triggered an adaptive response to counteract the stressor. In this case, enhancement of digestive functions could be observed^44,46^.

Insects, including *Acheta domesticus*, are recognized as valuable model organisms that enable research to be conducted in alignment with the 3R principle (replacement, reduction, and refinement). The sequencing of model insect genomes confirms their genetic and physiological similarities to other animals, including higher vertebrates. These advantages allow for the replacement of mammals in many experiments, particularly in the early stages of research. Insects can thus be used to investigate molecular mechanisms related to nutrition, and study dietary additives for their toxicity or health benefits. Despite species-specific differences, insects serve as robust models that can contribute to understanding human physiology, including nutrition, the genesis of metabolic disorders, and the functioning of the gut microbiome. Consequently, this study may provide universal insights applicable to other insect species and organisms, including humans^47,48^.

This study aimed to describe the effects of GO-AgNP composite nanomaterial on the model insect *Acheta domesticus* digestive functions examined from biochemical (activity of selected digestive enzymes), physiological (estimating food/energy budget parameters), and histological (identifying changes in the gut ultrastructure) perspectives. The study considered four different concentrations of GO-AgNP composite and length of exposure times. The tested hypothesis assumed that the possible changes in measured parameters of digestive functions would be proportional to the concentration of GO-AgNP composite and/or might intensify with the length of exposure time.

## Materials and methods

### Preparation and characterization of GO-AgNP composite

Graphene Oxide Water Dispersion (GO, 99.5%, 2 wt%) was purchased from Nano Graphen (Ankara, Turkey), and Silver Dispersion (Ag, 99.99%, 15 nm, 10000 ppm in water, Tawny) was obtained from US Research Nanomaterials, inc. (3302 Twing leaf Ln, Houston, TX 77084, USA).

By combining graphene oxide and silver nanoparticles with deionized water and citrate buffer solution (0.1 M, pH = 6.5), graphene oxide-silver nanoparticle composite suspension (GO200/Ag400 ppm NPs) was produced. Briefly, 24 mL of silver stock suspension, 6 mL of graphene oxide stock suspension and 100 mL of citrate buffer 0.1 M were pooled, adjusted to final volume with 300 mL of deionized water and sonicated (UP-100 H, DONSERV, Poland) for 4 h (cycle 1, amplitude 100% with gentle heating) and kept overnight in the dark at a room temperature to obtain final homogenous and stable colloidal suspension as described elsewhere^49–51^.

Four batches of feed were prepared with increasing content of GO-AgNP composite. 1.5, 15 and 150 mL of the composite suspension were diluted to a final volume of 150 mL with deionized water. Then, each solution was thoroughly mixed with 300 g of finely ground rabbit pellets to obtain dry-weight feed containing GO2Ag4, GO20Ag40 and GO200Ag400 ppm, respectively. For the highest NPs content in the feed (GO200Ag4000 ppm) a mixture of 120 mL of AgNPs and 3 mL of GO stock solutions was prepared and used similarly. Only deionized water (150 mL) was used for control feed preparation. Prepared feed was sterilized for 48 h in a laminar chamber (UVcleaner, BIOSAN, Warren, MI, USA) and dried for 48 h at 45 °C in a dryer (Pol-Eko Aparatura, Poland) (see Refs.^30,36^ for the detailed protocol of food preparation). The composite concentrations were selected based on our previous study^44^, in which we applied a single composite concentration (20 µg GO/g of food and 400 µg AgNPs/g of food) and observed signs of hormesis. To gain a deeper understanding of the digestive responses of *Acheta domesticus* to the composite and to determine whether it has adverse effects, we decided to expand the concentration range in the present study. The concentrations used ranged from 2 µg GO/g of food and 4 µg AgNPs/g of food to 200 µg GO/g of food and 4000 µg AgNPs/g of food.A scanning electron microscope (SEM) with an energy dispersion X-ray spectrometer (EDX) (Quanta FEG 250; FEI, Oregon, USA) and atomic force microscopy (AFM) (Agilent 5500) were used for the analysis of the shape and structure of the GO-AgNP composites. A more detailed description of the sample preparation method for analysis was previously published^44,53^.

**Fig. 1:**
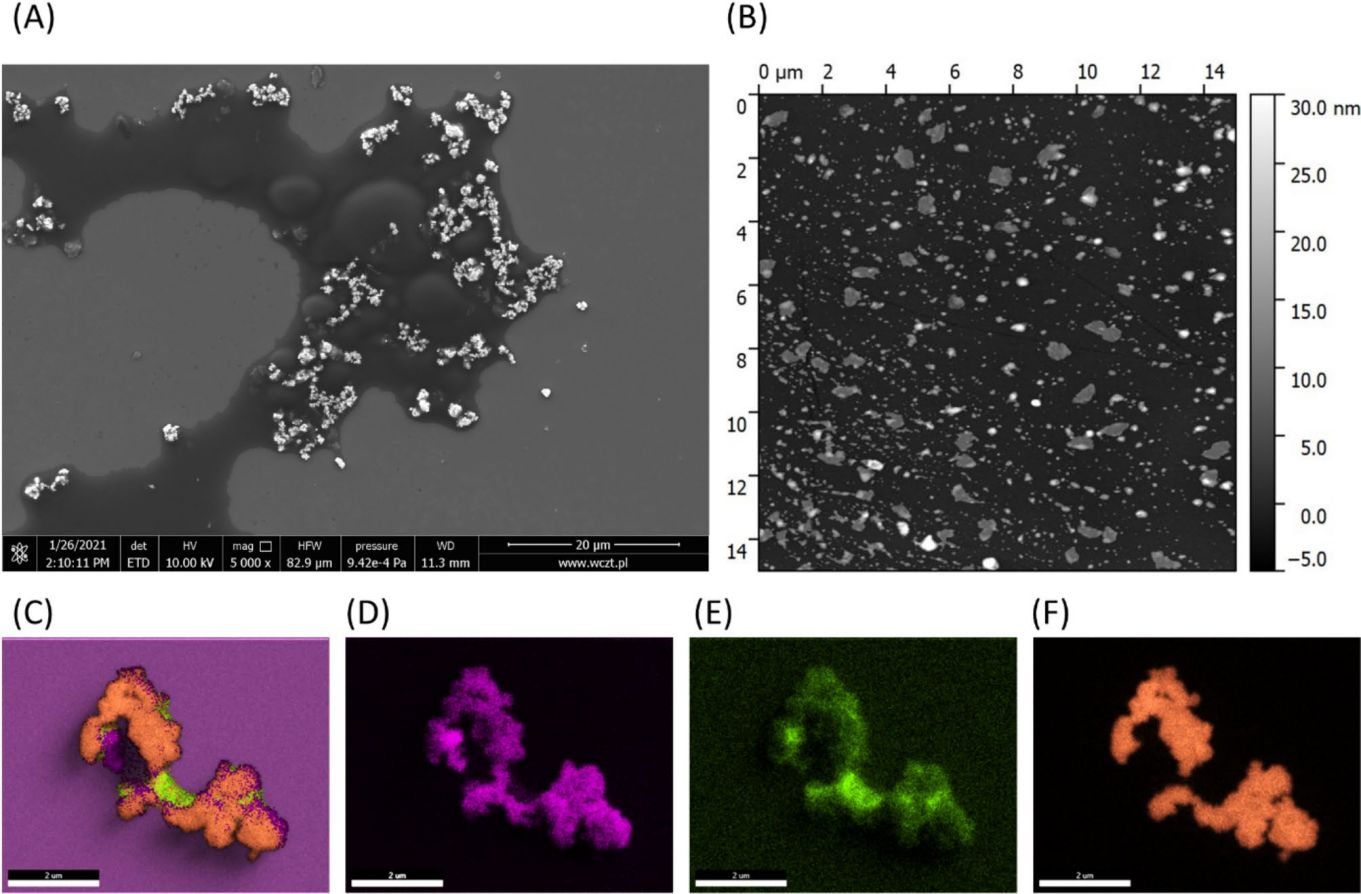
Physicochemical characteristics of GO-Ag NPs composite. (**A**) SEM, (**B**) AFM imagies. (**C**) SEM-EDS elemental mapping of GO-AgNPs composite with representative particles: (**D**) carbon, (**E**) oxygen, and (**F**) silver.

The GO-AgNP composite consisted of thick graphene oxide (GO) structures uniformly coated with silver nanoparticles. The average diameter of the nanoparticles was approximately 2 μm. Aggregates were observed sporadically, with diameters reaching 20–30 μm. The height of the structures varied between 1 and 30 nm (Fig. 1).

### House cricket (*Acheta domesticus*)

The model organism *Acheta domesticus* (Orthoptera, Insecta) has been used for decades in physiological and toxicological studies^54,55^. Its relatively short life cycle (approximately two months) enables observing the effects of studied compounds across multiple generations in a relatively short period. A high reproductive rate allows the establishment of numerous research groups and a satisfactory number of repetitions. Moreover, crickets are edible in some parts of the world and recently house cricket has been approved by European Commission as a novel food ingredient for humans^56^. Hence, studying the possible adverse effects of new environmental contaminants in the crickets becomes of interest to human health. The insects for the experiments were obtained from a permanent laboratory stock colony held at the Institute of Biology, Biotechnology, and Environmental Protection (Faculty of Natural Sciences, University of Silesia in Katowice, Poland)^57^.

### Experimental design

The 0–1 day-old adults from the stock cohort were randomly assigned to five experimental groups: control and fed with food containing GO-AgNP composite at the following concentrations: GO2Ag4, GO20Ag40, GO200Ag400, and GO200Ag4000 µg/g of food, prepared as described above. The insects were kept in plastic fauna boxes throughout the experiment, with free access to food, water, and shelter. Three distinct sets of insect groups were established for experimental purposes: (a) food budget assessment, (b) cell status analysis, and (c) enzymatic assays and histological analysis.

### Food consumption and assimilation measurements

Each experimental group had five replicates (boxes) containing five individuals in each box. For the first ten days, the dry weight of the supplied food, food remains, faeces, and the insects’ fresh weight were measured every two days, and then on the 16th and 21st day with a 1 mg accuracy (Semi-Micro Balance EX225D; OHAUS, Parsippany, NJ, USA). The dry weight of the samples was obtained by drying them at 50 °C for 48 h. These raw data were used to calculate food budget parameters in the experimental groups (see Refs.^44,45^ for a detailed description of budget calculations).

### Cell status analysis

The percentage of dead cells and oxidative stress in the *A. domesticus* gut was measured on the treatment’s first, fifth, and 21st days. Five insects from each group were randomly picked up and slightly anaesthetized on ice. Subsequently, the whole gut was excised and placed in 0.1 M PBS buffer (400 µL, 4 °C, pH 7.4). The tissue was then gently homogenized (Minilys, Bertin Technologies, France), and cytometry (Muse Cell Analyzer, Millipore, MA, USA) was used to identify the percentage of dead cells and oxidative stress in the resulting cell suspension, according to the protocol for Muse Annexin V & Dead Cell Kit and Muse Oxidative Stress Kit.

### Digestive enzyme measurements

Gut samples were taken on the 1st, 5th, 15th, and 21st days of the treatment from anaesthetized crickets by isolation and homogenization of the midgut in a phosphate buffer (pH 7.4, 1 mL, 4 °C), followed by centrifugation of the homogenates for ten minutes at 4 °C at 14,000 rpm. The midguts of three insects were pooled for each sample, weighing 100 ± 20 mg, and five replicates were made for every time point in each experimental group. The activity of digestive enzymes was assayed with commercially purchased kits following the provided protocols previously optimized for *A. domesticus*^45^. Carbohydrates are a key energy source for the house cricket, which is why we selected four enzymes involved in their digestion: α- and β-glucosidases (α-Glu, β-Glu), Amylase, and β-galactosidase (β-Gal). We also included enzymes digesting proteins and lipids to gain a comprehensive understanding of the composite’s effects.

Proteolytic activity was measured spectrophotometrically as changes in absorbance at 492 nm per minute using the Protease Assay Kit (Cat. No. 539125; LOT 3802816) from Calbiochem; Merck KGaA, Darmstadt, Germany.

Amylase activity was assayed with the Amylase Activity Assay Kit (Sigma-Aldrich, St. Louis, MO, USA; Cat. No. MAK009; LOT 8E24K07110) and expressed in µmol of product/min/mL supernatant.

Activity of α- and β-glucosidases (assay kits from Sigma-Aldrich, St. Louis, MO, USA; Cat. No. MAK123; LOT 123CA05A04 and Cat. No. MAK129; LOT 129CB08A18, respectively) were measured spectrophotometrically (TECAN Infinite M200, Männedorf, Austria) in 96-well flat-bottom plates, as the substrate-specific product formation reaction rate at 405 nm and expressed in Units/L (one unit represent the quantity of enzyme required to hydrolyze one µmol of substrate per minute).

Activity of β-galactosidase was measured with β-Galactosidase Activity Assay Kit (Abcam, Cambridge, CB2 0AX, UK; Cat. No. ab287846; LOT GR3429797-1). The reaction rate was determined spectrofluorimetrically for 20 min (Ex/Em = 480/530 nm using a Hitachi F-7000 Fluorescence Spectrometer Plate Reader, Tokyo, Japan) and expressed in Units/L, where 1 unit is the quantity of enzyme needed to produce 1.0 µmol of fluorescein per minute^58,59^.

The Lipase Activity Assay Kit (Sigma-Aldrich, St. Louis, MO, USA; Cat. No. MAK046; LOT 8H15K07220) was used to measure lipase activity with glycerol as the reference standard of product formation rate at 570 nm. The expressed value of enzyme activity was µmol/min/mL.

### Histological analysis

For this analysis, samples were taken from treated adult insects on the 5th and 21st days of exposure. The midgut was isolated and prepared for transmission electron microscopy (TEM) according to the protocol described by Karpeta-Kaczmarek et al.^60,61^. Ultra-thin Sect. (70 nm) were cut on a Leica Ultracut UCT25 and contrasted with uranyl acetate and lead citrate. Tissues were analyzed using a Hitachi H500 transmission electron microscope at 75 kV. The applied technique allows the visualization of cellular structures but not the presumed intracellular localization of the composite.

### Statistical analysis

Digestive enzymes activities and food budget analyses included five replicates. Before statistical data processing, Dixon’s Q test was applied to identify and reject outliers. Assumptions of the analysis of variance were checked for the obtained data (the Kolmogorov–Smirnov and Lilliefors tests to assess the data distribution and Levene and Brown–Forsythe tests to evaluate the homogeneity of variances) before further analysis.

Multivariate repeated measures ANOVA with Tukey post-hoc test (*p* < 0.05) was applied to assess the effects of NPs concentration and treatment time (= adult age) on food consumption and assimilation, cumulative food consumption and assimilation in crickets, as well as enzyme activity. The main effects of concentration and time, as well as their interactions, were also assessed.

## Results

### Food budget

**Fig. 2:**
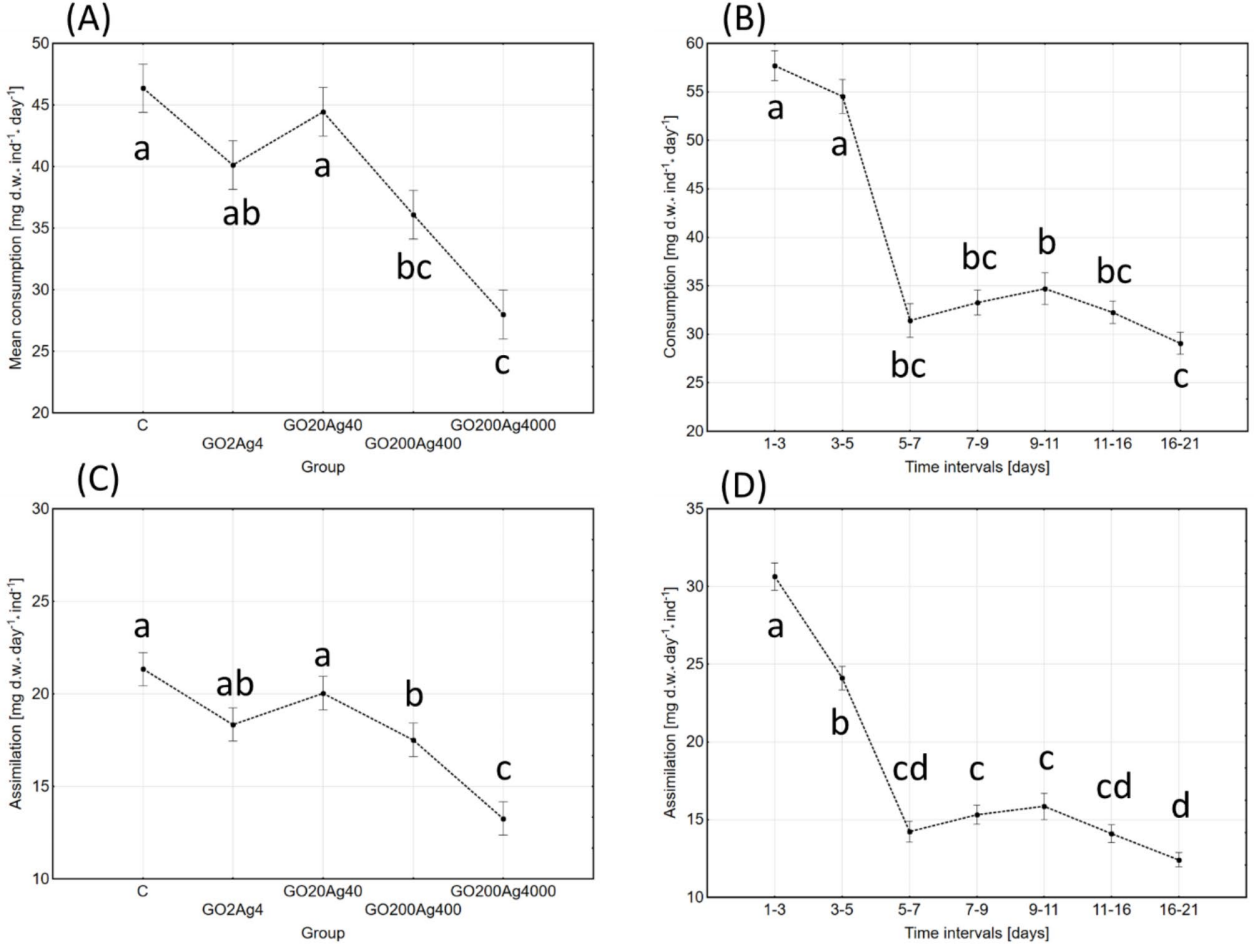
Effect of group (**A**,**C**) and time-intervals (**B**,**D**) on food consumption (**A**,**B**) and assimilation (**C**,**D**) (mg dry weight per individual per day; marginal means ± SE) in adult *A. domesticus* treated with different concentrations of GO-AgNPs. The same letters denote no significant differences among groups (ANOVA (**A** and** C**) or ANOVA with repeated measurements (B and D); Tukey post-hoc test; *p* < 0.05).

Food consumption in control was the highest during the first five days (reaching almost 72 mg dry weight food per individual per day), then lowered significantly in subsequent time intervals to a relatively similar level. The mean daily consumption in this group through 21-day interval (and age-span) was 42.70 mg. Exposure to NPs revealed a significant inhibitory effect of their concentration and exposure time. The joint effect of these factors was also highly significant (Table 1; Fig. 2). The lowest consumption was observed in the group GO200Ag4000 at all time intervals, with the mean daily value reaching hardly 64% of the control (27.36 mg d.w. per individual). The highest difference was observed in 1–3 and 3–5 days intervals when consumption reached 53% and 40% of the control values, respectively (see Fig S1 in Supplementary materials).

**Table 1:** Multivariate repeated measures ANOVA for GO-AgNP concentration [1], time [2], and interaction of the factors [1] × [2] on food consumption and assimilation measured in consecutive time (age) intervals within 21 days of adult crickets exposure.

Effect	Food consumption				Food assimilation			
	F	df_1_	df_2_	*p*	F	df_1_	df_2_	*p*
Concentration [1]	13.90	4	20	< 10^− 4^	11.75	4	20	< 10^− 5^
Time (age) [2]	85.63	6	120	< 10^− 5^	118.02	6	120	< 10^− 5^
[1] × [2]	3.81	24	120	< 10^− 5^	3.78	24	120	< 10^− 5^

Symbols description: F - F ratio; df_1_ and df_2_ - treatment and error degrees of freedom, respectively; p - p value, *n* = 5.

**Fig. 3:**
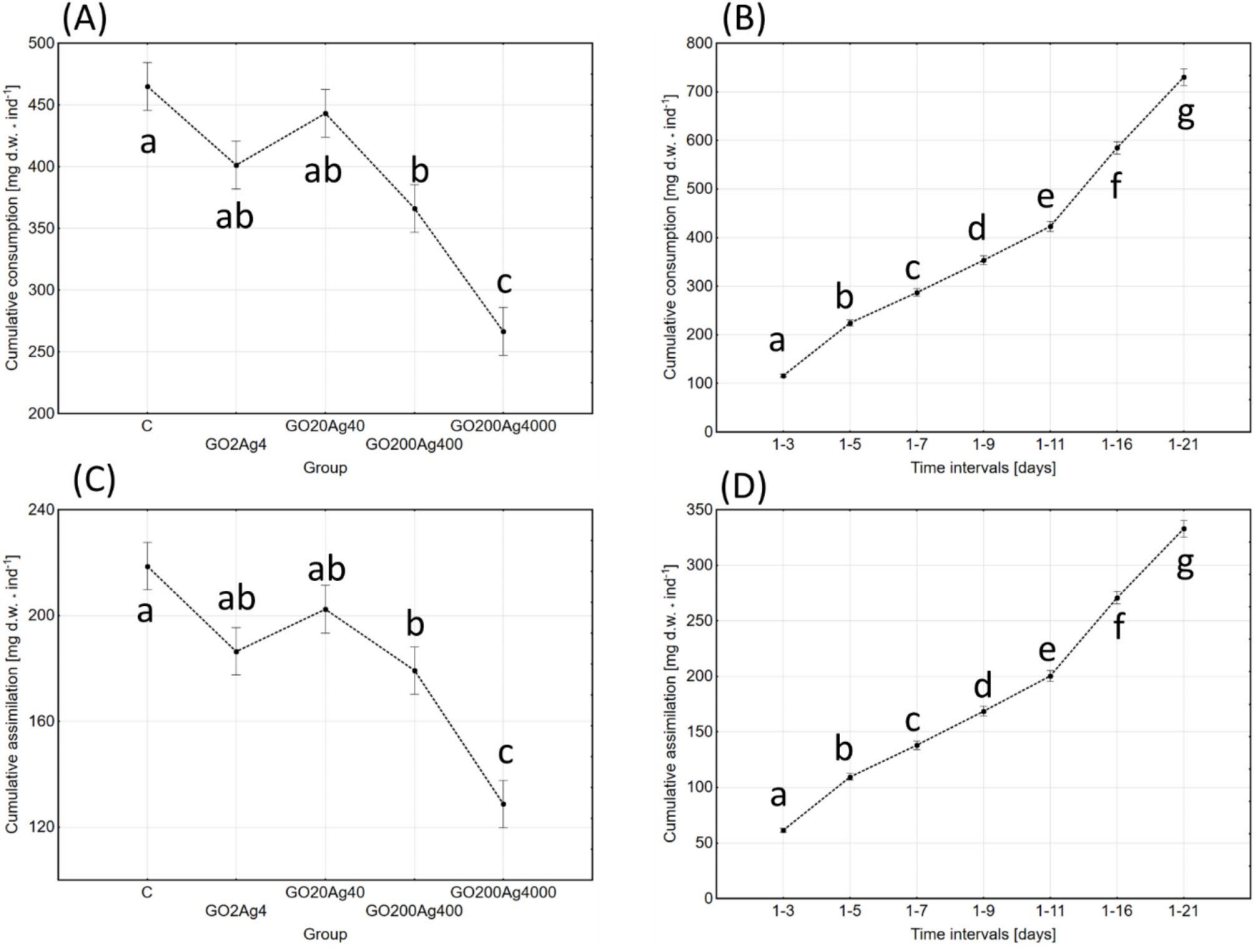
Effect of group (**A**,**C**) and time-intervals (**B**,**D**) on cumulative food consumption (**A**,**B**) and cumulative food assimilation (**C**,**D**) (mg dry weight per individual per day; marginal means ± SE) in adult *A. domesticus* treated with different concentrations of GO-AgNPs. Abbreviations: see Fig. 2.

Cumulative food consumption (CFC) across the analysed period of adult life revealed similar and even higher significance of NPs-concentration and time-intervals (age) effects in comparison with daily consumption data (Table 2; Fig. 3, and Fig S3 in Supplementary materials).

**Table 2:** Multivariate repeated measures ANOVA for GO-AgNP concentration [1], time [2], and interaction of the factors [1] × [2] on cumulative food consumption (CFC) and cumulative food assimilation (CFA) measured in consecutive time (age) intervals within 21 days of adult crickets exposure. Symbols used as in Table 1.

Effect	CFC				CFA			
	F	df_1_	df_2_	*p*	F	df_1_	df_2_	*p*
Concentration [1]	16.43	4	20	< 10^− 5^	14.33	4	20	< 10^− 4^
Time (Age) [2]	1192.60	6	120	< 10^− 6^	1284.10	6	120	< 10^− 6^
[1] x [2]	6.08	24	120	< 10^− 6^	4.54	24	120	< 10^− 6^

Assimilation of ingested food in the control was the highest during the first three days of adult life, and it was significantly lower in subsequent time intervals. The mean daily assimilation (calculated as weighted mean) for the analysed period was 19.30 mg of d.w. food per individual. Admixture of GO-AgNP composite in food lowered the assimilation, and both NPs concentration and time intervals, considered separately or jointly, had significant effects (Table 1). The lowest assimilation was observed in the group GO200Ag4000 at all time intervals, with the mean daily value reaching hardly 66% of the control (12.76 mg d.w. per individual). Following the consumption pattern, the most pronounced effect was observed during the first five days (see Fig S2 in Supplementary materials).

Cumulative food assimilation (CFA) followed the CFC pattern in control and experimental groups and confirmed the significance of previous calculations of concentration and time/age effects (Table 2 and Fig S4 in Supplementary materials). It is noteworthy that assimilation efficiency was similar in all the groups and reached values within 44.3–47.8% range. This indicates that NPs primarily affected food consumption, which caused observed changes in its assimilation.

Lowered consumption and assimilation resulted in smaller weight gain of adults throughout the experimental period (F = 3,458, df_1_ = 4, *p* = 0.0265) (Figs S5-S6 in Supplementary materials).

### Gut cell status

**Fig. 4:**
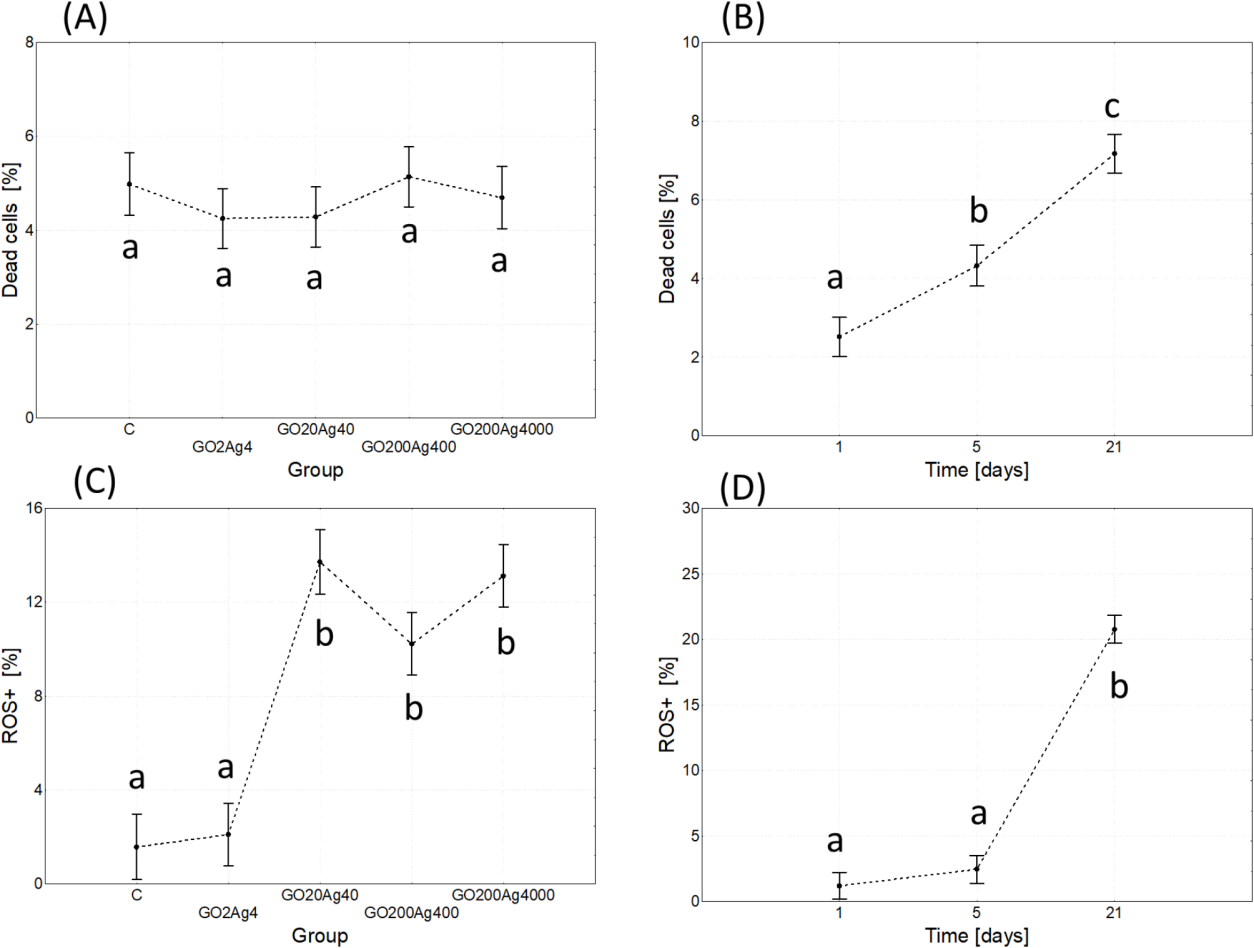
Effect of group (**A**,**C**) and time (**B**,**D**) on dead cells (**A**,**B**) and ROS + cells (**C**,**D**) (%; marginal means ± SE) in the gut of adult *A. domesticus* treated with different concentrations of GO-AgNPs. Abbreviations: see Fig. 2.

The main effects analysis revealed that neither of the used GO-AgNP composite concentrations influenced the percentage of dead cells. In contrast, the exposure time and the interactions between both variables were significant (Table 3; Fig. 4). The percentage of dead cells increased with the exposure time (age of the insects). This effect was particularly pronounced in the control group and the groups treated with lower concentrations of the GO-AgNP composite (GO2Ag4 and GO20Ag40). Interestingly, in the groups treated with higher concentrations of the composite, the percentage of dead cells over time did not change (in GO200Ag400 group) or even decrease (in GO200/Ag4000 group) (Fig. S7).

**Table 3:** The main effects and interactions of the factors: GO-AgNP concentration [1], time [2], and interaction of the factors [1] × [2] on dead cells ratio and oxidative stress level measured in the gut of *A. domesticus* at 1st, 5th and 21st days of exposure.

Effect	Dead cells			Oxidative stress		
	F	df	*p*	F	df	*p*
Concentration [1]	0.39	4	0.815	19.00	4	< 0.001
Time (age) [2]	22.56	2	< 0.001	109.05	2	< 0.001
[1] x [2]	9.93	8	< 0.001	19.60	8	< 0.001
Residual		58			58	

See Table 1 for symbols description.

Both the variables of concentration and time and their interactions affected the level of oxidative stress in the gut cells of *A. domesticus* (Table 3). The ROS + level was significantly elevated in the groups treated with higher composite concentrations (GO20Ag40, GO200Ag400, GO200Ag4000) compared to the control and the GO2Ag4 group, which did not differ. Additionally, the ROS + level was significantly higher on day 21 compared to days 1 and 5 (Fig. 4). A more detailed analysis (Fig. S7) showed that this effect was due to the increase in ROS + in the groups treated with higher composite concentrations.

### Digestive enzyme activity

**Fig. 5:**
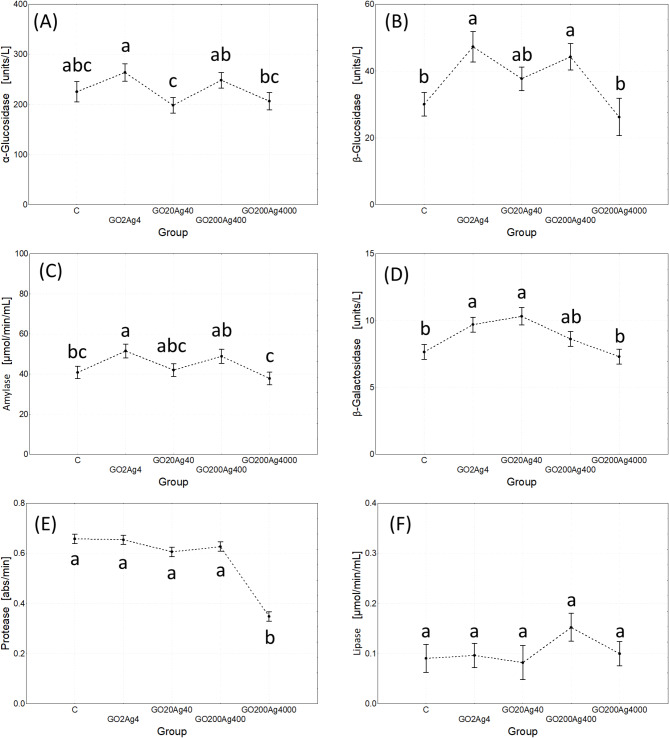
Effect of treatment (experimental group) on digestive enzyme activity (marginal means ± SE) in adult *A. domesticus* treated with different concentrations of GO-AgNPs. The same letters denotes no significant differences among groups (Tukey post-hoc test; *p* < 0.05).

Multivariate repeated measures ANOVA revealed an ambiguous effect of the composite concentration on the hydrolytic activity in the gut. It was significant only for β-Glu, β-Gal, amylase, and protease, and the first two enzymes had higher activity in composite NP-exposed cricket vs. control, except the highest composite concentration used. The activity of the assayed enzymes in the latter was as in control, and protease activity was the lowest (Table 4; Fig. 5).

**Fig. 6:**
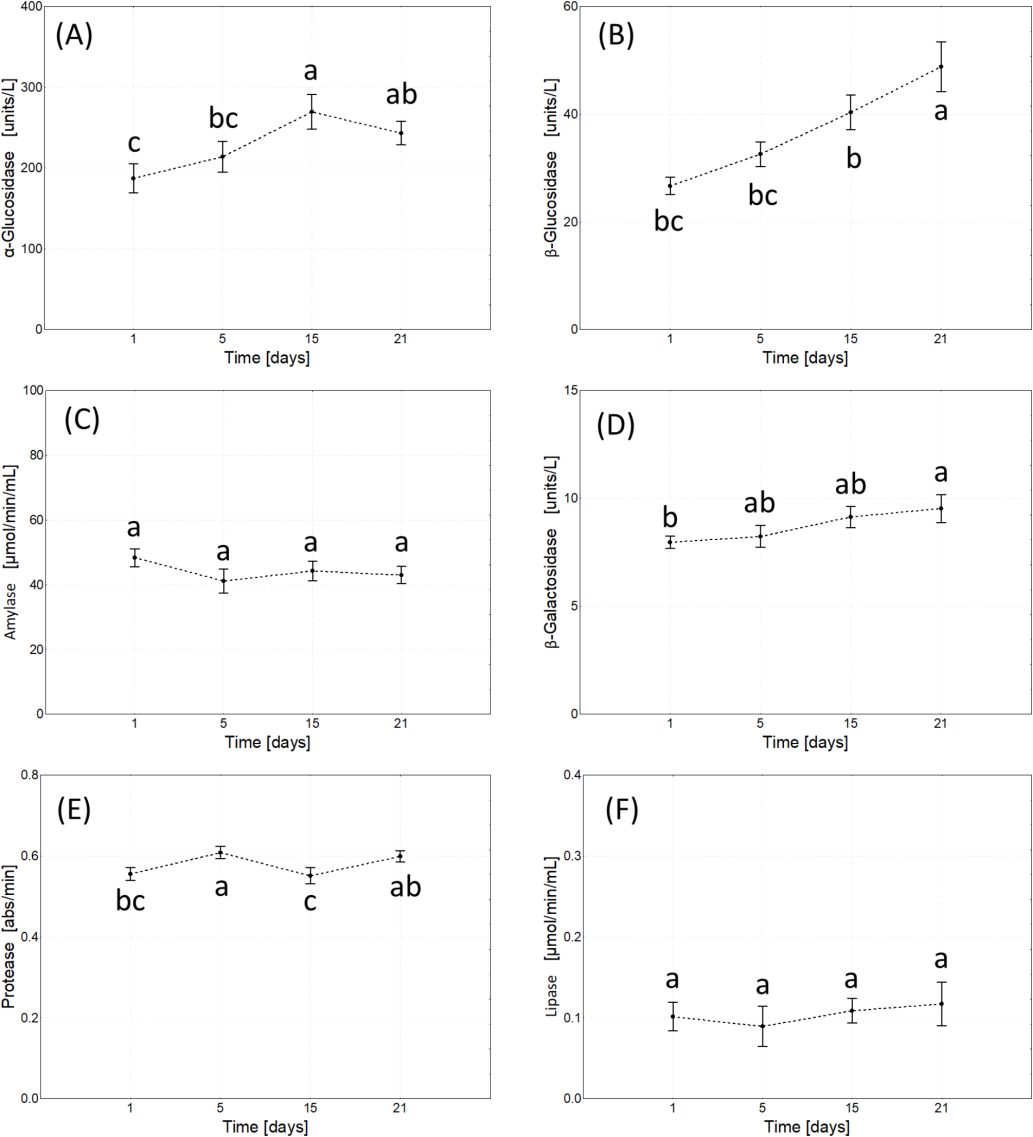
Effect treatment duration (time) on digestive enzyme activity (marginal means ± SE) in adult *A. domesticus* treated with different concentrations of GO-AgNPs. The same letters denotes no significant differences among groups (Tukey post-hoc test; *p* < 0.05).

With exposure time, the activity of glucosidases and protease increased, while other enzymes did not change their activity. However, concentration and exposure time interaction revealed the affected activity of all assayed enzymes except α-glucosidase (Table 4; Fig. 6 and S8).

**Table 4:** Multivariate repeated measures ANOVA for GO-AgNP concentration [1], time [2], and interaction of the factors [1] × [2] on digestive enzymes in the gut of *A. domesticus* following exposure to GO-AgNP composite, measured in consecutive time (age) within 21 days of adult crickets exposure (F - F ratio; df_1_ and df_2_ - treatment and error degrees of freedom, respectively; p - p value, *n* = 5).

Effect	F	df_1_	df_2_	*p*	F	df_1_	df_2_	*p*	F	df_1_	df_2_	*p*
	α-Glu				β-Glu				β-Gal			
Concentration [1]	2.702	4	16	0.068	3.987	4	14	0.023	4.839	4	14	0.012
Time [2]	3.478	3	48	0.023	11.295	3	42	< 0.001	2.265	2	42	0.095
[1] × [2]	0.869	12	48	0.582	2.767	12	42	0.007	2.687	12	42	0.009
	Amylase				Lipase				Protease			
Concentration [1]	2.937	4	18	0.049	0.946	4	11	0.473	48.524	4	20	< 0.001
Time [2]	0.959	3	54	0.419	0.334	3	33	0.801	3.284	3	60	0.028
[1] × [2]	4.220	12	54	< 0.001	3.548	12	33	0.002	1.963	12	60	0.044

### Gut histology

**Fig. 7:**
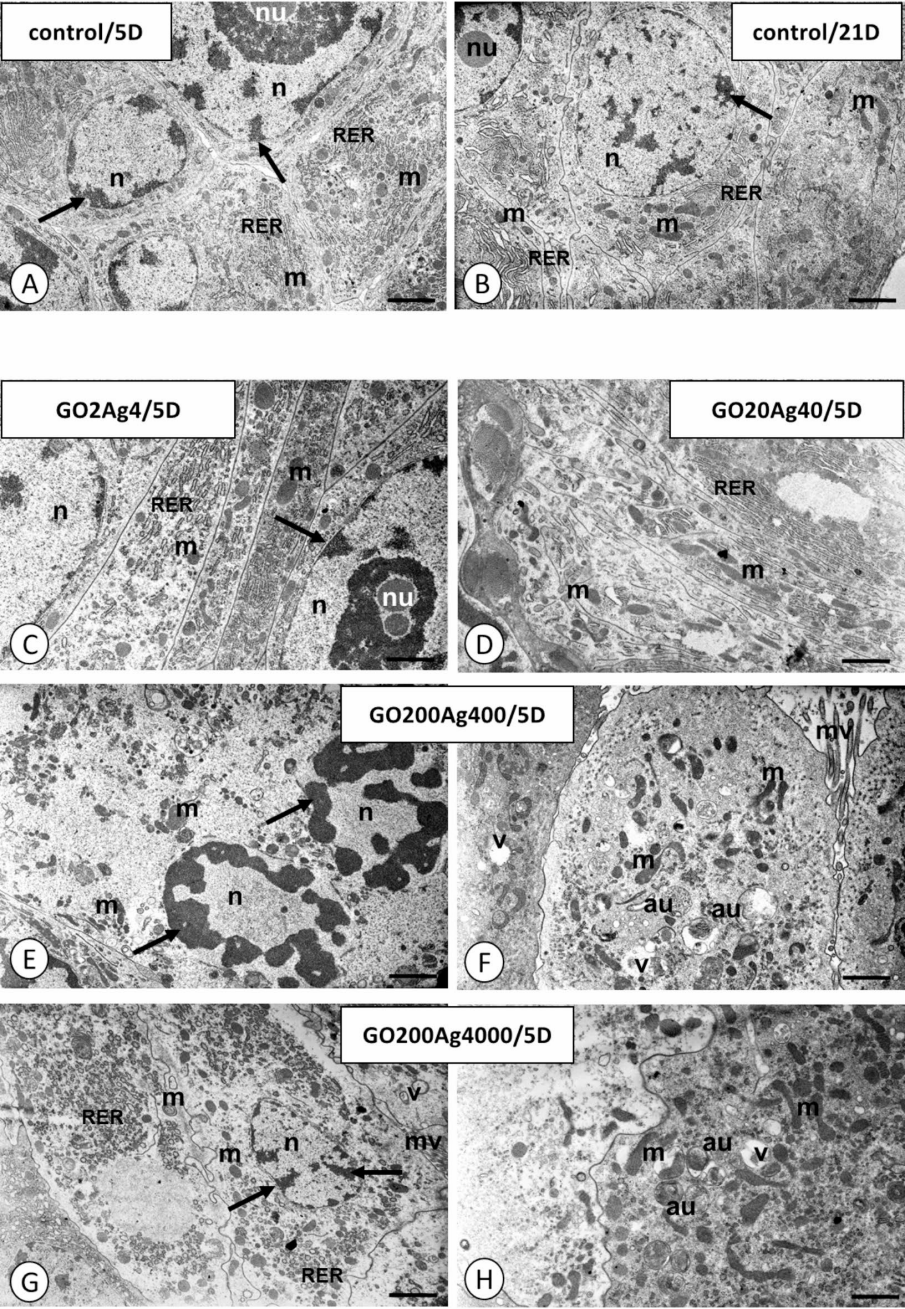
*Acheta domesticus* midgut epithelium in control (**A**,**B**) and experimental groups GO2Ag4 (**C**), GO20Ag40 (**D**), GO200Ag400 (**E**,**F**), and GO200Ag4000 (**G**,**H**) after 5 days (/5D) of exposure to GO-AgNPs. Mitochondria (m), nuclei (n), nucleoli (nu), patches of heterochromatin (arrows), cisterns of RER (RER), autophagic structures (au), vacuoles (v). TEM. (**A**) Scale bar = 0.8 μm. (**B**) Scale bar = 1.2 μm. (**C**) Scale bar = 0.9 μm. (**D**) Scale bar = 0.7 μm. (**E**) Scale bar = 1 μm. (**F**) Scale bar = 0.9 μm. (**G**) Scale bar = 0.9 μm. (**H**) Scale bar = 0.7 μm.

The midgut of *A. domesticus* comprises two distinct regions: the anterior and posterior parts. It is lined with the simple epithelium formed by two types of cells: the digestive and regenerative cells (midgut stem cells), which form characteristic regenerative crypts. In the five- and 21-day-old control crickets, three regions could be distinguished in the cytoplasm of the digestive cells: basal, perinuclear, and apical, while the cytoplasm of regenerative cells was poor in organelles and did not show any regionalization in organelles distribution (Figs. 7A-B). This description is consistent with earlier analysis of digestive and regenerative cells ultrastructure^58,59^.

**Fig. 8:**
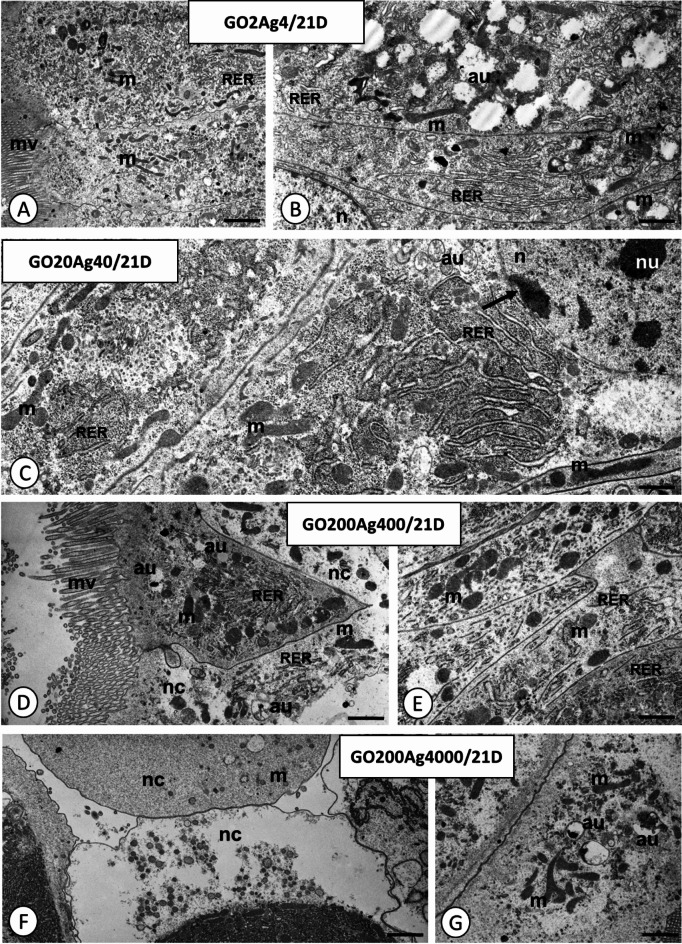
*Acheta domesticus* midgut epithelium in experimental groups GO2Ag4 (**A**,**B**), GO20Ag40 (**C**), GO200Ag400 (**D**,**E**), and GO200Ag4000 (**F**,**G**) after 21 (/21D) days of exposure to GO-AgNPs. Mitochondria (m), nuclei (n), nucleoli (nu), patches of heterochromatin (arrows), cisterns of RER (RER), autophagic structures (au), necrotic cells (nc). TEM. (**A**) Scale bar = 1.5 μm. (**B**) Scale bar = 0.7 μm. (**C**) Scale bar = 0.5 μm. (**D**) Scale bar = 0.9 μm. (**E**) Scale bar = 0.7 μm. (F) Scale bar = 1.6 μm. (**G**) Scale bar = 0.9 μm.

In five-day-old crickets from GO2Ag4 and GO20Ag40 groups, no changes were observed in the digestive and regenerative cells of the midgut epithelium (Figs. 7C-D). In crickets of the same age from GO200Ag400 and GO200Ag4000 groups, the apical cytoplasm of digestive cells often showed necrotic features: the cytoplasm was electron-lucent and poor in organelles. Vacuoles with electron-lucent interiors and autophagic structures appeared, and many mitochondria were damaged (Figs. 7E-H). In the perinuclear cytoplasm, disrupted nuclear envelopes could be observed. The chromatin formed electron-dense patches in numerous digestive cells (Fig. 7E). The cytoplasm of regenerative cells showed no changes compared to the control group. Similar changes were observed only in the digestive cells of 21-day-old individuals (GO2Ag4, GO20Ag40, GO200Ag400, and GO200Ag4000; Figs. 8A-G) compared to 5-day-old adult crickets. However, more autophagic structures (autophagosomes, autolysosomes, residual bodies) could be observed in all these experimental groups (Fig. 8B-D and G). In addition, necrosis was intensified in the cells of 21-day-old individuals in GO200Ag400 and GO200Ag4000 groups. Thus, cells with electron-lucent cytoplasm and poor organelles appeared, and the apical cell membrane lost the microvilli (Fig. 8D and F).

## Discussion

Despite extensive research on the toxicity of GO and AgNPs, studies on GO-AgNP composites remain limited, particularly regarding their effects on the digestive functions of exposed animals. Our previous study^44^ with a single concentration of GO-AgNPs composite has shown weak or moderate stimulatory response in food digestion and absorption, similar to the effects of these NPs applied separately. Observed changes resembled stress-induced adaptive reactions.

The current study explores previous observations in the same model insect (*Acheta domesticus*) by broadening the composite concentration range and elongation of the exposure time, almost to the mean adults’ survival time, that is 22.4 ± 5.2 days for both sexes (unpublished data). Results indicated that food consumption and assimilation depended significantly on both concentration and exposure time (Table 1; Fig. 2). While GO-AgNPs did not disrupt the typical feeding patterns of *A. domesticus*, (except for the group receiving the highest composite concentration, Figs S1-S2), cumulative consumption increased less towards the end of the feeding period across all treated groups (Fig. S3). Sparse relevant literature data suggests potential impairments of digestive functions and supports our findings^62–67^. Regarding nutrients and energy assimilation, no significant differences were observed in protein, lipid, glucose, and glycogen levels in *Gammarus roeseli* exposed to silica nanoparticles (30–1000 nm). However, the authors ascribed a lack of significant differences between groups to small sample sizes^68^. A 21-day exposure of *A. domesticus* to GO alone showed no impact on cumulative food consumption and a moderate, yet significant, increase in food assimilation at concentration of 200 µg/g food. In contrast, exposure to AgNPs at concentrations of 4, 40, and 400 µg/g food tended to decrease both parameters, although the effect was not concentration-dependent. High concentrations of AgNPs may promote their aggregation and clustering, thus reducing the total surface area of NPs and Ag ions release^45^. Hence, it seems reasonable to suppose that the observed concentration-dependent effects of the GO-AgNP composite were caused merely by AgNPs. GO flakes probably maintained the dispersion and stabilization of silver nanoparticles by preventing their agglomeration^69^.

The behavior of the GO-AgNP composite in the gut, specifically whether it aggregates or dissociates into GO and AgNPs, remains unclear. Nanoparticles interact with nutrients, host-derived compounds, and gut microbiota. Changing conditions within the gut, including pH fluctuations, enzyme concentrations, and the formation of new compounds, may influence nanoparticle stability. However, evidence from prior studies suggests that the GO-AgNP composite exhibits high stability. Zhu et al.^70^ attributed its sustained antibacterial activity to the exceptional stability of its nanostructures. Similarly, Bao et al.^35^ highlighted the hydrophilicity and stability of GO, which effectively prevent composite aggregation and AgNP dissociation.

Possibly stable, the composite’s particles in the gut milieu may cause damage to epithelial cells. The number of dead cells and cells with elevated oxidative stress (ROS+) increased with cricket’s age (Figs. 4B and D) and also in one-day-old individuals exposed to higher concentrations of the composite (Fig. S7). Interestingly, in the latter, the percentage of the dead cells did not change with adults’ age. This finding suggests a possible link to cell cycle arrest, as seen in various cells exposed to different NPs^71,72^. Observed increased ROS + cells at higher composite concentrations (Fig. S7B) may suggest their elevated molecular damage, including DNA damage. The arrest of the cell cycle, increasing the likelihood of repairing damage and preventing its irreversibility could be potential cellular response to such stress^73^. Such cells may re-enter the cell cycle, potentially reducing the number of dead cells. In case of nutrient deprivation (e.g. following decreased food consumption caused by xenobiotic’s presence), cell cycle arrest may save energy resources and avoid cell death^74,75^. Moreover, it was-documented that cell cycle arrest can postpone cellular senescence with retaining metabolic activity for an extended time^76^. This may contribute to decreased number of dead cells in the high-stress exposed tissue, compared to the control. Following these assumptions, it could be hypothesized that higher NPs composite concentrations cause cellular damage, while cell cycle arrest provides a window for damage repair or adaptation to stressful conditions. A similar delay in the progression of percentage of dead cells, typical of aging individuals, was observed in crickets exposed to AgNPs at concentrations of 4 and 40 µg/g of food and GO^45^.

We previously showed that despite DNA damage, increased ROS + cells, and induced apoptosis, GO can cause adverse histological changes in the midgut of *A. domesticus*^77^. Symptoms of autophagy or necrosis in insects’ still-living cells exposed to high GO-AgNP composite concentrations (Figs. 7 and 8) suggest activation of defense mechanisms, attempts at cell repair, or initiation of programmed cell death. While the mechanism of GO-AgNPs action in intestinal cells is unknown, recent studies by Feng et al.^78^ revealed that GO toxicity in these cells involves the p53 protein. They also observed structural damage to small intestine cells in GO-exposed rats, suggesting that the adverse effects of oral GO exposure are significant^78^. Additionally, GO can cut mitochondrial membranes causing organelle damage and impairing ATP production^79^. The toxicity mechanism of AgNPs is also linked to the p53 protein, cell cycle arrest, and apoptosis induction^71,80–82^. We can hypothesize on these fragmentary data that both components of GO-AgNP composite exert similar effects by increasing ROS production, weakening the cell’s antioxidant defenses, arresting the cell cycle, and then inducing programmed cell death or, potentially, cell regeneration. A similar mechanism for GO-AgNPs was postulated in studies on human liver normal (CHANG) and cancer (HepG2) cells^83^, caprine fetal fibroblast cells^84^, and U87 cancer cell lines and also in the fungus *Alternaria alternata* cells^85^. However, at this research stage, we cannot confirm whether the GO-AgNP composite enters digestive and/or regenerative cells. Recent studies conducted by Lange et al.^25^ on bacterial cells suggest that the GO-AgNP composite may accumulate around cells or on their surface, primarily interacting with the cell wall and membrane. However, the authors of this study do not rule out other points of interaction with cells, including membrane damage, ROS generation, and inhibition of cell growth^25^. Apart from oxidative stress, other common symptoms included the accumulation of autophagosomes and autophagic vacuoles^84^. Furthermore, there is evidence that the nanocomposite’s cytotoxic effects are stronger than AgNPs and GO given separately^22^.

Disruptions in the cell cycle and gut cell structure caused by the GO-AgNP composite were accompanied by changes in digestive enzyme activities, albeit in a limited range. The main effects showed stimulation of some carbohydrate-degrading enzymes at low nanocomposite concentrations and significant inhibition of protease at the highest concentration (Fig. 5). The joint effect of exposure time and treatment provided a more complex picture (Fig. S8). Crickets, like some other insects, can regulate protein and carbohydrate intake and metabolism^86^. Stimulation of carbohydrate-degrading enzyme activities, the most readily available energy source, may indicate a compensatory adjustment to meet increased energy demands under stress. Reduced protein utilization in the GO200Ag4000 group raises concerns, as protein is an essential source of amino acid for endogenous molecule synthesis and proper development^87,88^. However, in this study, impaired protein utilization in the GO200Ag4000 group did not significantly hinder fresh weight gain of adult crickets (Figs S5-S6), although it might have affected other functions related to protein synthesis (e.g. nutrients allocation to eggs production in females).

The mechanism underlying the observed effects of NPs composite on digestive enzymes remains enigmatic. However, stimulation of carbohydrate-degrading enzymes and inhibition of proteases have been noticed in earlier studies in various species, including insects exposed to AgNPs^45^. Studies by Muralisankar et al.^89^ demonstrated that the freshwater prawn *Macrobrachium rosenbergii* exhibited improved growth parameters, survival rates, and increased activity of digestive enzymes, including proteases, when dietary zinc nanoparticles (ZnNPs) were provided at concentrations up to 60 µg/g. However, exceeding this concentration resulted in toxic effects, including inhibiting digestive enzymes. Similarly, protease inhibition was observed in juvenile *Epinephelus coioides* exposed to copper nanoparticles (CuNPs) at concentrations of 0.02–0.1 µg/mL for 25 days^38^. The authors proposed that this inhibition might result from a direct effect of nanoparticles on enzyme activity or synthesis, ruling out changes in feeding behaviour, feeding activity, or food quality due to nanoparticle contamination.

We believe that insights into the interaction between nanoparticles and digestive enzymes come from research on nanocatalysis and the use of enzyme-NP conjugates in industry for rapid decomposition of complex molecules^90–92^. Although these studies focus mainly on the structures created in a laboratory (ex vivo), they shed light on the mechanisms of enzyme-NP conjugate formation and the role of NPs in modulating enzyme activity. For example, Deka et al.^91^ observed a nine-fold increase in α-amylase activity when a low concentration (0.175 µg/mL) of citrate-stabilized gold nanoparticles (cit-AuNPs) was added to the reaction milieu. Transmission electron microscopy (TEM) and dynamic light scattering (DLS) revealed enzyme molecules attached to cit-AuNPs. The α-amylase molecule, which contains two cysteine thiol groups away from the active site, bound to NPs, forming a complex with a more effectively oriented active center. However, these structures can form varying levels of NP agglomeration, affecting enzyme activity.

Following this, it seems reasonable that optimal NPs/enzyme molecules ratios may enhance enzymatic activity due to conformational changes, while higher NP concentrations forming larger agglomerates may reduce enzyme functional group availability. Saware et al.^92^ found that immobilizing α-amylase on gold nanoparticles and biosynthesized silver nanoparticles increased enzyme activity one- to two-fold compared to the ‘free’ enzyme.

Metal nanoparticles also affect other catalytic properties of digestive enzymes’^90,93,94^. For example, gold nanoparticles coated with PEG-biotin and lipase digested substrates differently than ‘free’ lipase, with significant dependency on enzyme orientation, decreasing activity when the active site faced the nanoparticle^90^. Therefore, NPs can act as catalysts within specific concentration ranges, facilitating enzyme-substrate interaction.

The above leads to a tentative conclusion that the observed activity of digestive enzymes following exposure to NPs composite may result from structural damage to the epithelial gut cells producing the enzyme molecules and conformational changes of these molecules caused by NPs that affect the activity. Moreover, in the gut milieu, NPs are surrounded by various nutrients, host-produced molecules, and the gut microbiome, which makes these molecular interactions far more complex.

## Conclusions

**Fig. 9:**
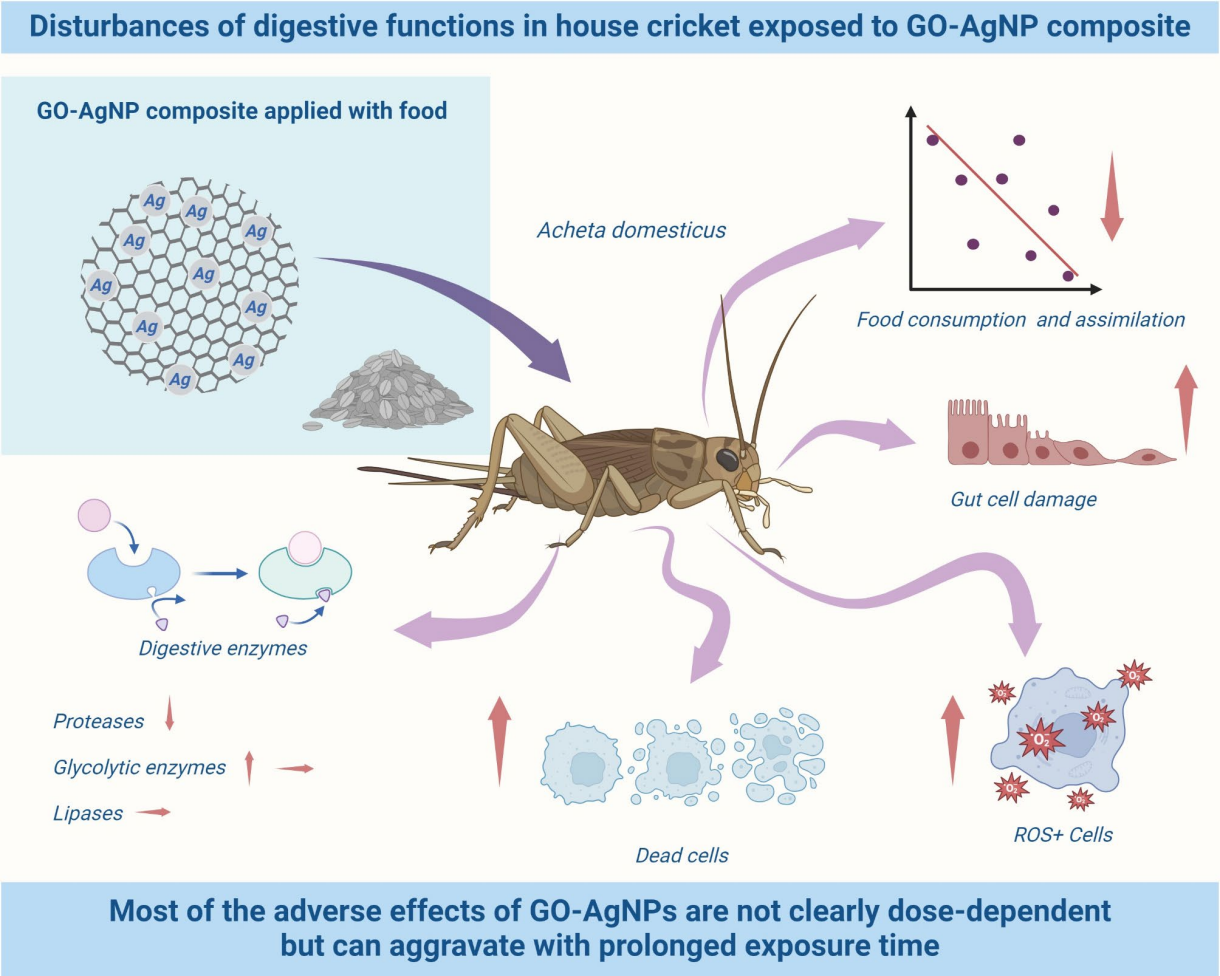
Disturbances of digestive functions in house cricket exposed to GO-AgNP composite.

The results of this study can support the hypothesis that the GO-AgNP composite can disrupt gut structure, digestive enzymes’ activity and food/energy budget parameters (Fig. 9). Some of these changes were not dose-dependent. However, they became more pronounced with prolonged exposure (age of organisms and indirectly with increasing composite concentration). Such results suggest complex interactions among composite NPs and target organism’s constituents. However, current state-of-the-art allows speculative rather than definitive explanations of their mechanisms, particularly concerning in vivo exposure. Nevertheless, our results point out that the composite of GO-AgNPs, like its constituents (described in our previous papers), cannot be considered harmless, particularly in higher concentrations and/or long-term exposure.

Our research has limitations, notably in understanding how the GO-AgNP composite affects digestive functions, including enzyme activity. Adding the composite to the feed and the required sterilization and drying processes may have affected its organoleptic properties and altered (slightly) its composition. Future studies should investigate the composite’s fate after ingestion, including whether its particles penetrate gut cells or if AgNPs dissociate in the gastrointestinal tract. Another interesting question to be studied is the role of gut microbiota in the NPs effects within the gut tissue and milieu.

## Electronic supplementary material

Below is the link to the electronic supplementary material.

Supplementary Material 1

## Data Availability

Raw data are provided on the RepOD database (DOI: 10.18150/XKOIEP; https://repod.icm.edu.pl/dataset.xhtml? persistentId=doi%3A10.18150%2FXKOIEP).
