# Supplementary material for: Concentration-dependent disturbances of digestive functions in house cricket (Insecta: Orthoptera) exposed to GO-AgNP composite
Source: Sci Rep. 2025 Apr 13;15:12699. doi: 10.1038/s41598-025-97589-w (PMC11994775; doi:10.1038/s41598-025-97589-w)
Supplement: Supplementary file 1 — Supplementary Material 1 [file 41598_2025_97589_MOESM1_ESM.docx]

**Supplementary Materials**


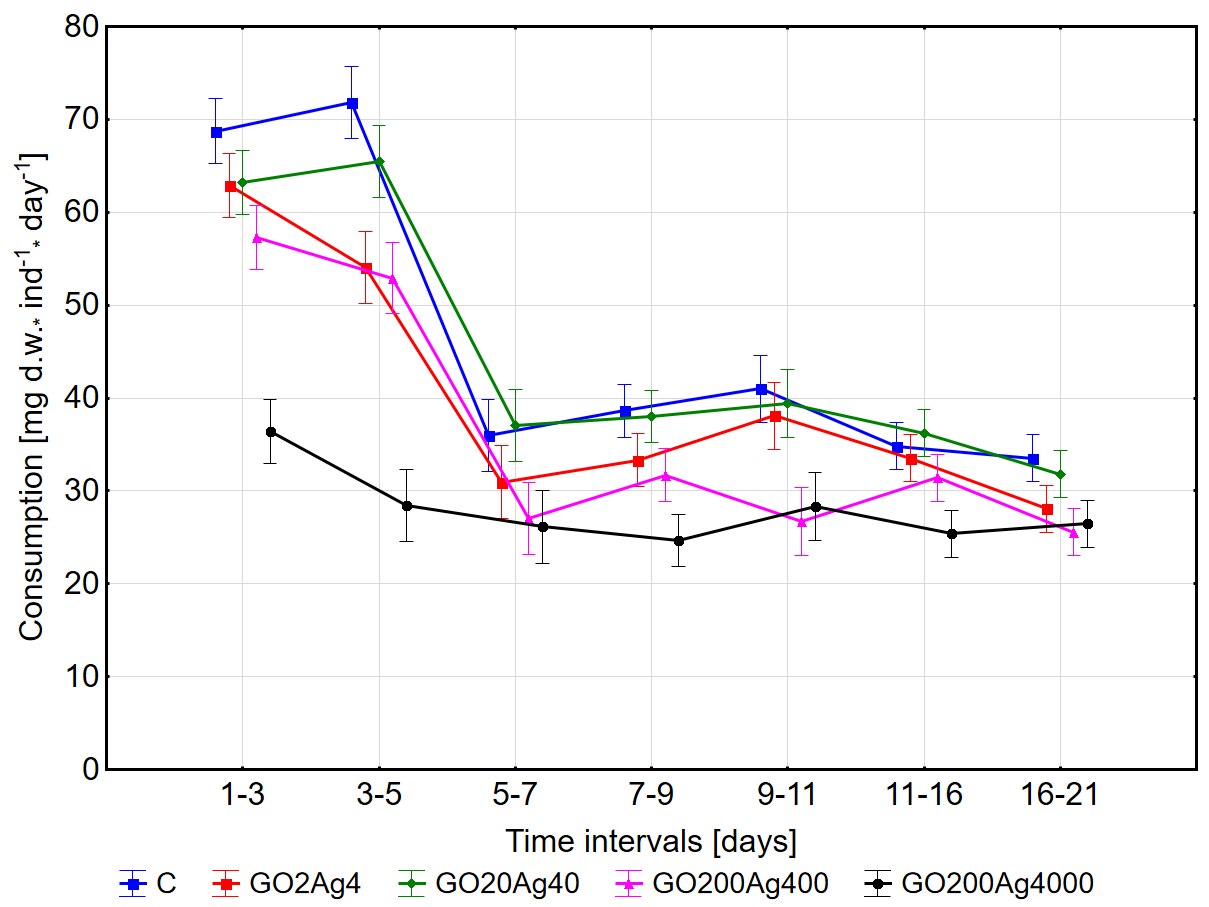


**Figure S1.** Food consumption (mg dry weight per individual per day; mean + SE) in consecutive time-intervals (days) by adult *A. domesticus* treated with different concentration of nanoparticles. See M&M for detailed groups description.


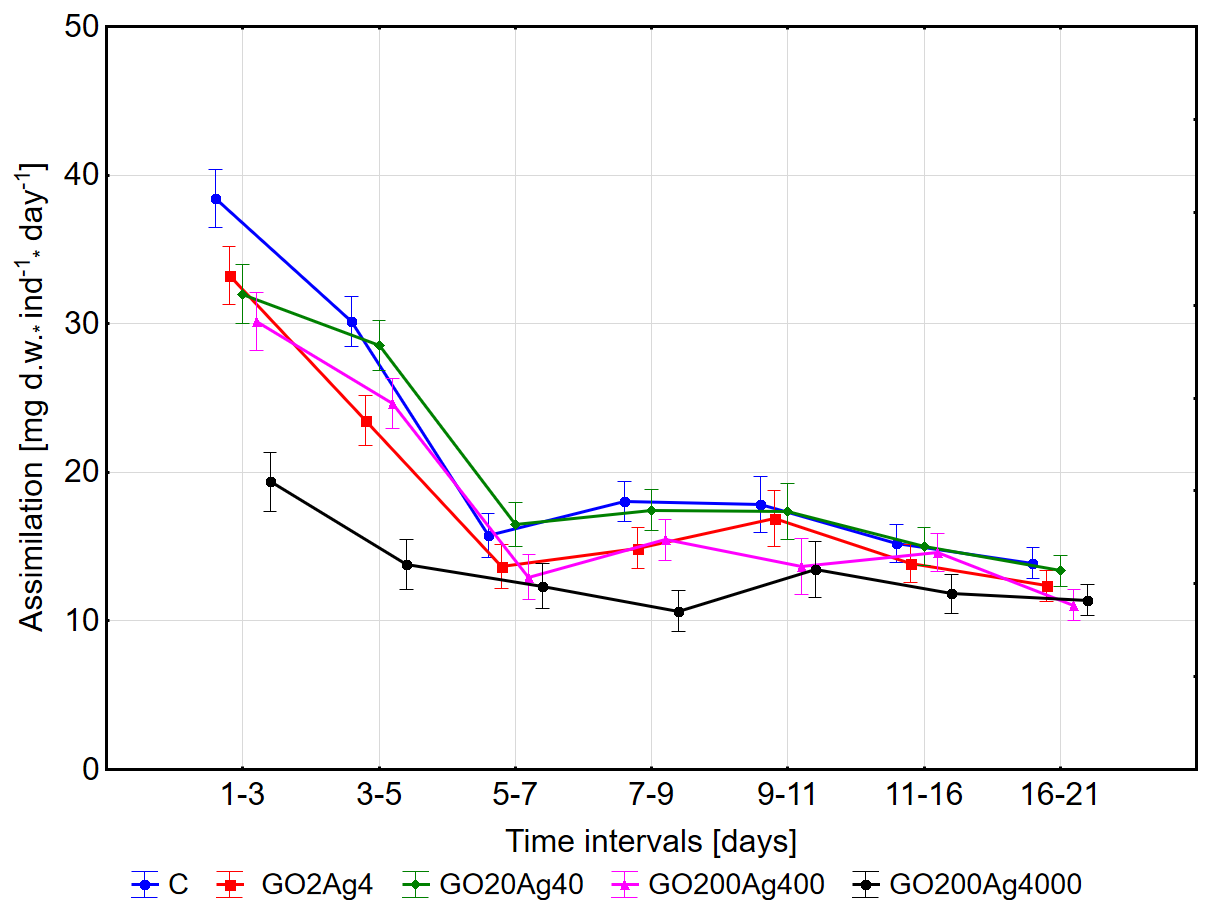


**Figure S2.** Food assimilation (mg dry weight per individual per day; mean + SE) in consecutive time-intervals (days) by adult *A. domesticus* treated with different concentration of nanoparticles. See M&M for detailed groups description.


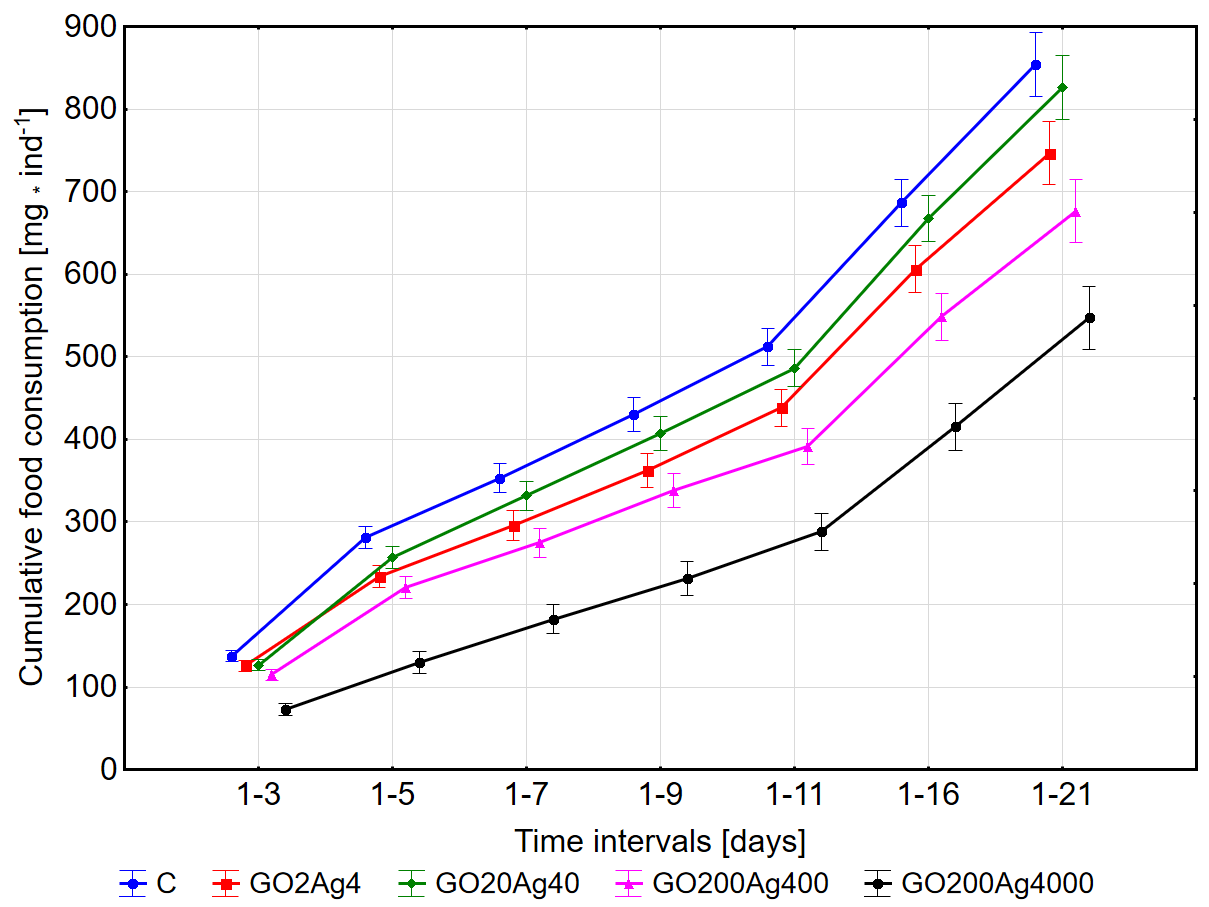


**Figure S3**. Cumulative food consumption (CFC, mg dry weight per individual per day; mean + SE) in consecutive time-intervals (days) by adult *A. domesticus* treated with different concentration of nanoparticles. See M&M for detailed groups description.


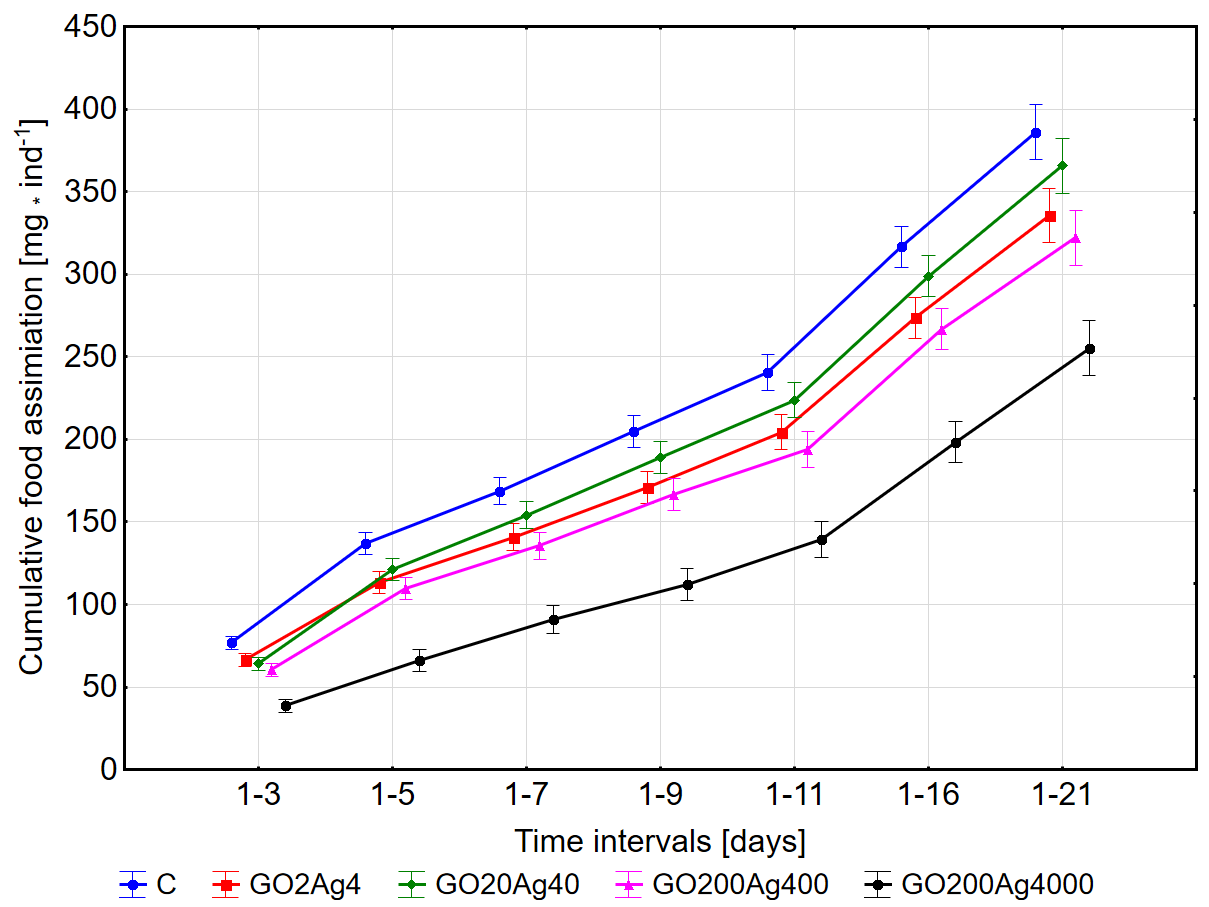


**Figure S4.** Cumulative food assimilation (CFA, mg dry weight per individual per day; mean + SE) in consecutive time-intervals (days) by adult *A. domesticus* treated with different concentration of nanoparticles. See M&M for detailed groups description.


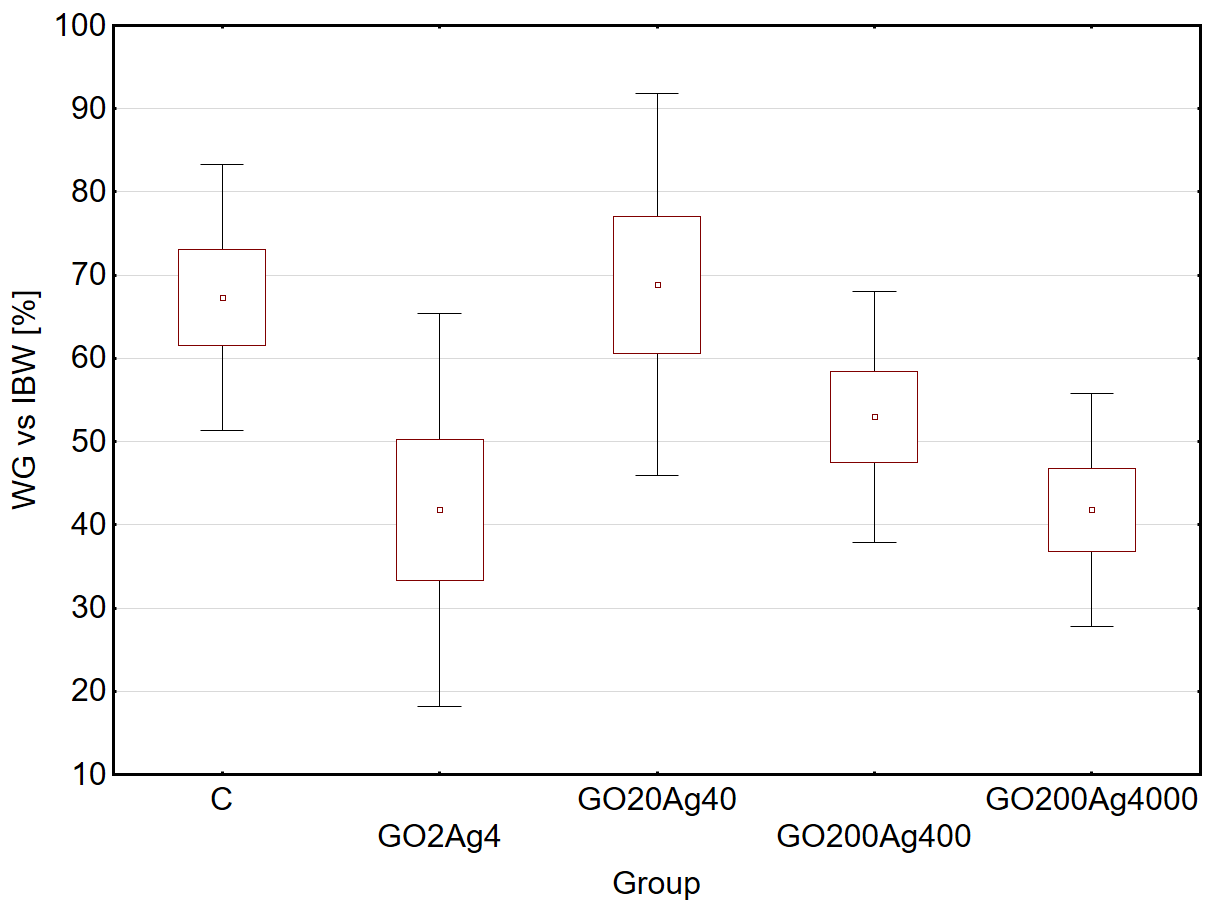


**Figure S5.** Percentage of fresh weight gain (WG) relative to initial weight (IBW) of adult crickets during the experiment in the control and groups treated with different concentration of GO-AgNPs.


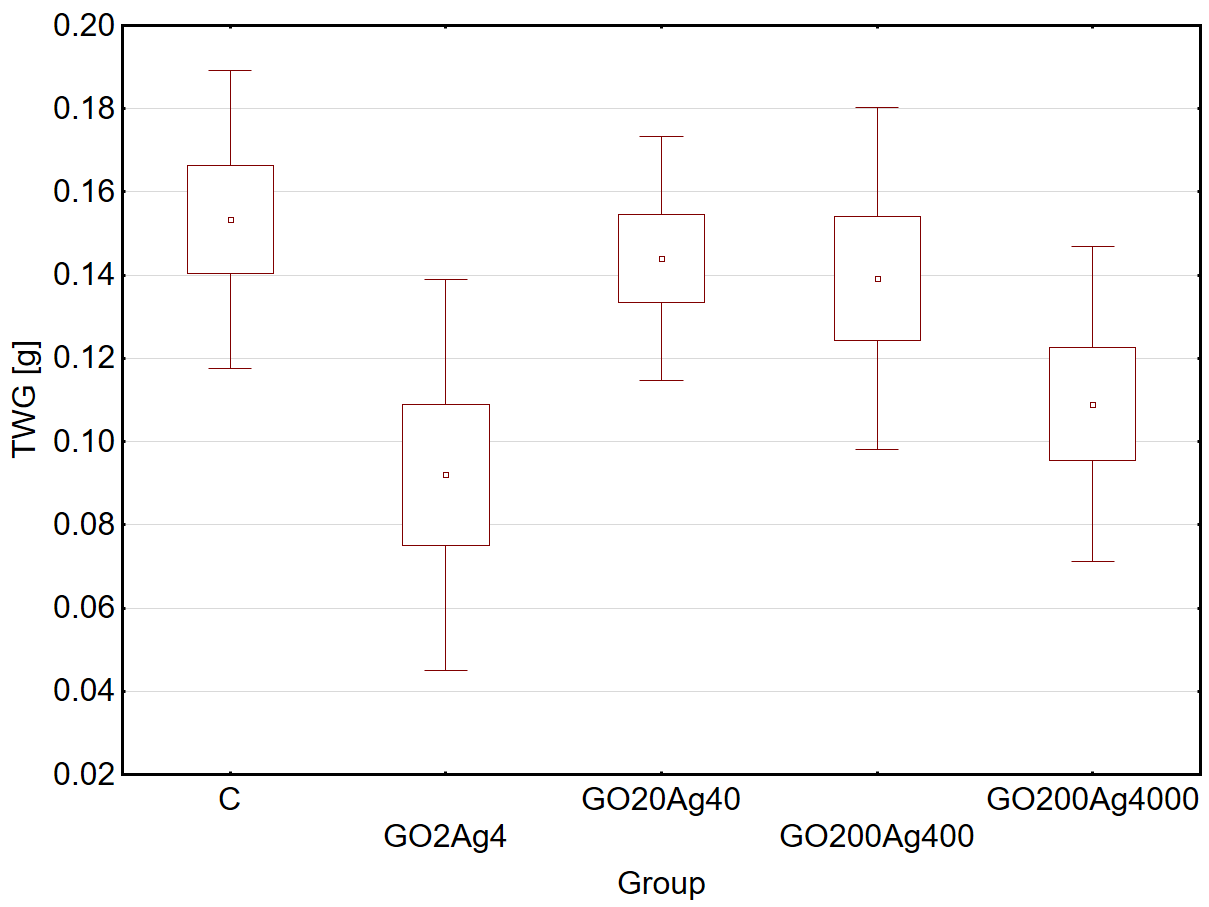


**Figure S6.** Total fresh weight gain [g] in adult crickets from the control and experimental groups groups treated with different concentration of GO-AgNPs.


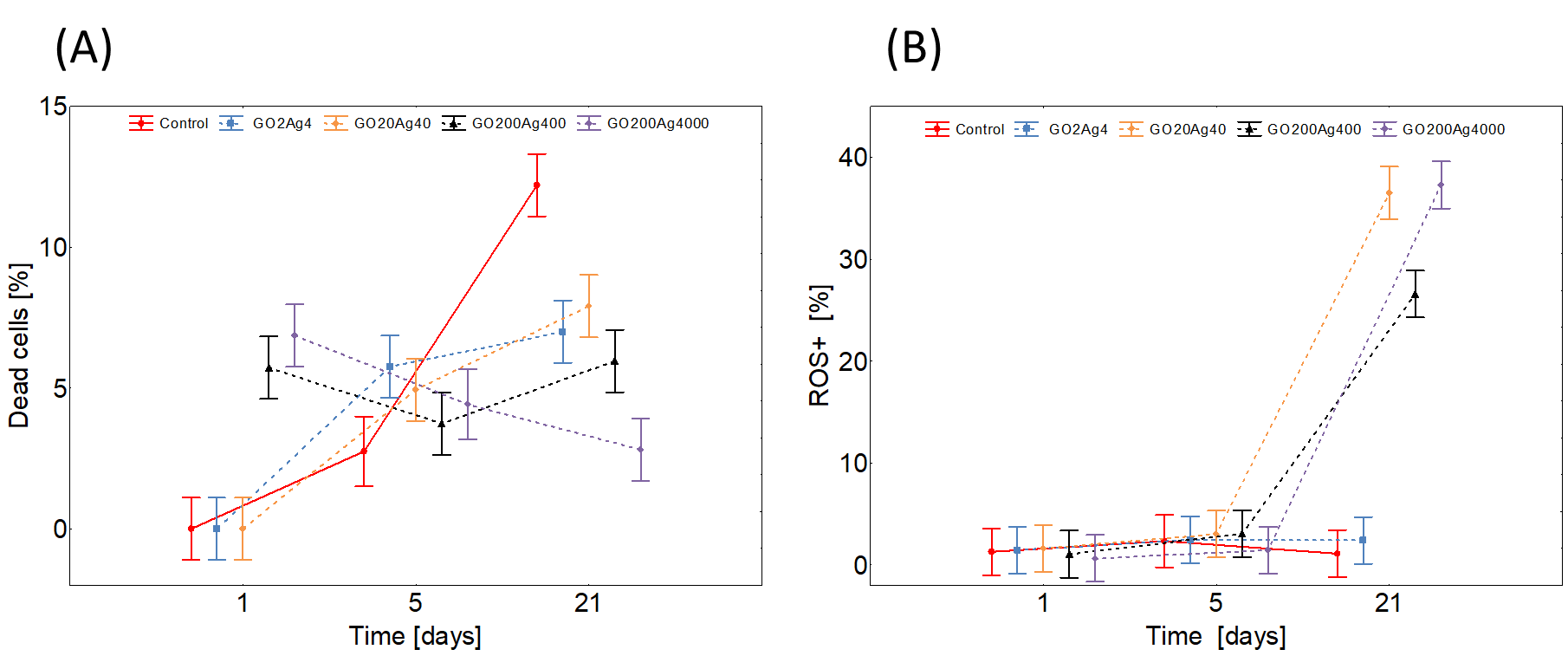


**Figure S7.** Dead cells (A) and ROS+ cells (B) (%; mean ± SE) in the gut of adult *A. domesticus* treated with different concentrations of GO-AgNPs. See M&M for detailed groups description.


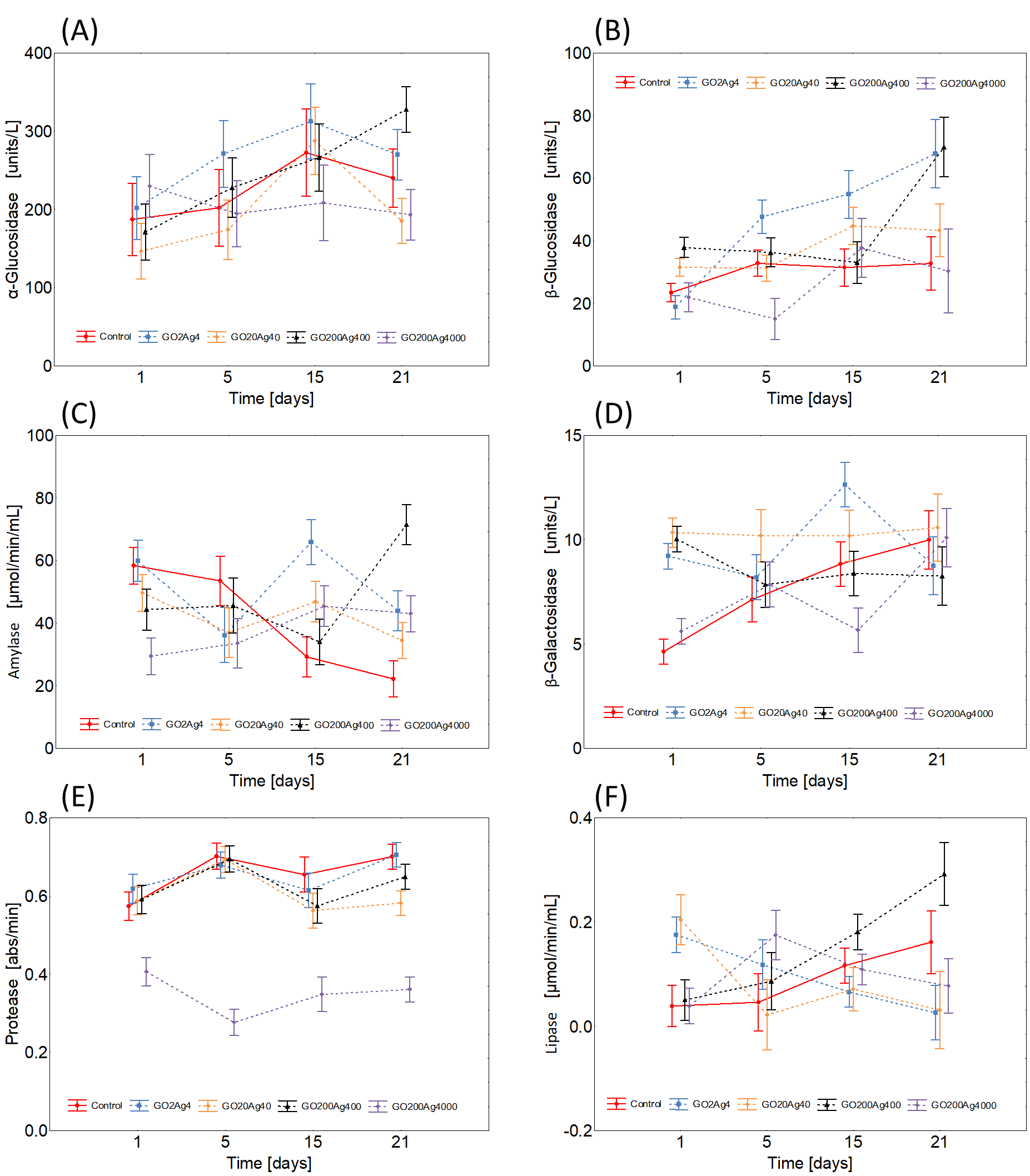


**Figure S8**. Digestive enzyme activity: (A) α-Glucosidase; (B) β-Glucosidase, (C) Amylase, (D) β-Galactosidase, (E) Protease, and (F) Lipase (mean ± SE) in the gut of adult *A. domesticus* treated with different concentrations of GO-AgNPs. See M&M for detailed groups description.
